# Objectives of Sustainable Development and Non Transmissible Chronic Disease

**DOI:** 10.1590/1518-8345.0000.2717

**Published:** 2016-06-14

**Authors:** Lorena Chaparro-Díaz

**Affiliations:** Lorena Chaparro Díaz is Associate External Editor of Revista Latino-Americana de Enfermagem, Associate Professor of Nursing College, Universidad Nacional de Colombia, Colombia and President of Capitulo Upsilon Nu, Sigma Theta Tau International. Email: olchaparrod@unal.edu.co

The Agenda Post 2015 to sustainable development proposes the 17 Objectives of Sustainable Development (ODS) that will be the focus of world interest in the next 15 years. Nursing cannot ignore this milestone of global work that will significantly contribute to improve the quality of life of people, families and communities. The International Council of Nursing and Society of Nursing Honor Sigma Theta Tau International (STTI) acknowledged that Nursing has a important role to success of those targets. STTI fostered a strategy to give a voice to women and nursing at United Nations, with active participation in world decisions in health subject and consolidated a work group led by the nurse Holly Shaw, from New York^1^ ^).^

**Figure:**
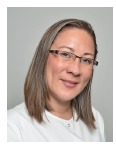

It is undeniable that objective number 3 "To assure a healthy life and foster the well being of all people in all ages" is the one of greatest emphasis in our subject; However, there are other challenges in the other 16 objectives. This text intends to reflect about those objectives and the innovative care of nursing in Non Transmissible Chronic Disease (ECNT) from the experience I had in the research group nursing care to chronic patient ^(^ ^2^. 

Objective. ECNT is closely related to poverty, as it is one of causes of risk factors to ECNT. However, when there is a chronic conditions, there are factors that generate poverty in families related to home care of person with ECNT upon existing a greater demand of public services, necessity of taking over transport costs and using family economical resources to assure the opportunity of health assistance with additional services that are not provided by assistance plans in the countries or access essential medicines of treatments. 

Objective 4. The family caregivers of people with ECNT and also people in situations of incapacity have few possibilities of accessing advanced education. A proof of it are the low indexes of education and gender inequality. The care work in ECNT is delegated to informal caregivers with or without minimum education to read and write, which is a risk to patient's health. 

Objective 5 and 8. The care economics (non remunerated care) estimated a great contribution, specially of women, to financial sustainability of countries with "Care of life at home". But it was not socially and politically recognized when it was not esteemed and rewarded upon laws. In the same way, the migration has made that the informal care work is a possibility of personal economic development, but it endangers the health of caregivers and also of the person who is being cared because there is no assurance to this work of a minimum education and a professional and institutional continuity. 

On the other hand, the access to decent work of people with ECNT, in situation of mental or physical incapacity and to family care is limited by incompatibility in times and spaces that are still traditional in some areas. 

Objective 10. The first world countries have important developments in care areas at ECNT. But it are the networks and research allegiances that generate valid scientific knowledge that will enable to contribute to inequality on countries and among the same. The instruction and leadership programs that are provided in scientific organizations, like STTI can be a important strategy to favor equality^3^. 

Objective 11. The health systems still have access barriers to services with small appropriation of Information Technologies and communication through strategies of Telehealth and Telecare. On the other hand, the domicile assistance programs and of home assistance are more and more limited to vulnerable populations due to bad infrastructure, reduced access to safe transport and unsafe and inaccessible neighborhoods. 

Objective 12. To nursing, the culture of garbage degradation is something very common in the approach to bio safety. But, upon transferring the care of chronicity to the home, there is a important challenge of this culture related to the education of patient and his caregivers, comparing a hospital scenery that integrates the reduction, recycling and reuse, thus, attempting to reduce the intangible costs in health. 

Objective 13. The climatic change is a reality. However, from preventing ECNT it is feasible to contribute and impact on individual and family level in the short term and on collective level in the long term. An example is the culture of riding a bicycle, which has the individual challenge to adopt a good lifestyle, a family benefit with the reduction of transport costs and collective benefit with contributing to reduce the emissions of carbon monoxide of traditional means of transport. 

Objective 16. Taking care of a person with ECNT, either a child, adult or elderly person means a important sacrifice of the family, where only a special connection of the caregiver (family caregiver -person with ECNT) ^(^ ^4^ will allow his maintenance in care giving. It is very alarming to find cases of abandonment and bad treatment, specially in elderly adults in conditions of high dependence. The culture of care among generations was lost and it is required to resume the commitment of children and reduce the load of care in ECNT or rethink other assistance models. 

Those nine objectives mean a reflection of lines and research programs of groups, resumes and nursing colleges. It does not mean to publish alone a isolated research result, but showing how it is contributed to a global sustainability with care innovations. Each strict and valid scientific contribution will enable the consolidation of original contributions of nursing to health of the world and improve the conditions of nurses upon being socially and politically acknowledged. I invite the readers to deepen each work area, so that it is contributed to quality of life through those objectives in the next 15 years. 
